# High levels of carbonic anhydrase IX in tumour tissue and plasma are biomarkers of poor prognostic in patients with non-small cell lung cancer

**DOI:** 10.1038/sj.bjc.6605690

**Published:** 2010-05-11

**Authors:** M İlie, N M Mazure, V Hofman, R E Ammadi, C Ortholan, C Bonnetaud, K Havet, N Venissac, B Mograbi, J Mouroux, J Pouysségur, P Hofman

**Affiliations:** 1 Laboratory of Clinical and Experimental Pathology, Louis Pasteur Hospital, Nice, France; 2 INSERM ERI-21/EA 4319, Faculty of Medicine, University of Nice Sophia Antipolis, Nice, France; 3 University of Nice Sophia Antipolis, Nice, France; 4 Institute of Developmental Biology and Cancer Research, University of Nice Sophia Antipolis and CNRS, UMR 6543, Nice, France; 5 Human Tissue Biobank Unit/CRB INSERM, Louis Pasteur Hospital, Nice, France; 6 Department of Mathematical Sciences, University of Nice Sophia Antipolis, Nice, France; 7 Department of Radiotherapy, Antoine-Lacassagne Cancer Center, Nice, France; 8 Department of Thoracic Surgery, Louis Pasteur Hospital, Nice, France

**Keywords:** carbonic anhydrase IX, non-small cell lung cancer, tissue microarray, plasma, prognosis

## Abstract

**Background:**

Carbonic anhydrase IX (CAIX) is an enzyme upregulated by hypoxia during tumour development and progression. This study was conducted to assess if the expression of CAIX in tumour tissue and/or plasma can be a prognostic factor in patients with non-small cell lung cancer (NSCLC).

**Methods:**

Tissue microarrays containing 555 NSCLC tissue samples were generated for quantification of CAIX expression. The plasma level of CAIX was determined by ELISA in 209 of these NSCLC patients and in 58 healthy individuals. The CAIX tissue immunostaining and plasma levels were correlated with clinicopathological factors and patient outcome.

**Results:**

CAIX tissue overexpression correlated with shorter overall survival (OS) (*P*=0.05) and disease-specific survival (DSS) of patients (*P*=0.002). The CAIX plasma level was significantly higher in patients with NSCLC than in healthy individuals (*P*<0.001). A high level of CAIX in the plasma of patients was associated with shorter OS (*P*<0.001) and DSS (*P*<0.001), mostly in early stage I+II NSCLC. Multivariate Cox analyses revealed that high CAIX tissue expression (*P*=0.002) was a factor of poor prognosis in patients with resectable NSCLC. In addition, a high CAIX plasma level was an independent variable predicting poor OS (*P*<0.001) in patients with NSCLC.

**Conclusion:**

High expression of CAIX in tumour tissue is a predictor of worse survival, and a high CAIX plasma level is an independent prognostic biomarker in patients with NSCLC, in particular in early-stage I+II carcinomas.

Non-small cell lung cancer (NSCLC) comprises approximately 80% of lung cancers, and nearly 50% of patients with stage I NSCLC die within 10 years of diagnosis (Travis *et al*, 2004; Ou *et al*, 2007). Despite major advances in surgical techniques and new strategies of neoadjuvant treatment, long-term survival is achieved in only 5–10% of NSCLC patients (Stinchcombe and Socinski, 2009). In this regard, early biomarkers are urgently needed to allow better clinical management of patients with NSCLC.

Hypoxia is common in various types of solid cancers and is associated with a more aggressive phenotype (Brahimi-Horn *et al*, 2007). Stabilisation of the α-subunit of the hypoxia-inducible transcription factor 1 (HIF-1*α*) is a critical step in the adaptation of tumour cells to hypoxia (Pouyssegur *et al*, 2006). One of the consequences of HIF-1 activation is upregulation of glycolysis and hence the production of lactic acid (Pastorekova *et al*, 2004). The enzyme carbonic anhydrase IX (CAIX), which is expressed on the tumour cell surface, catalyses the hydration of cell-generated carbon dioxide into protons and bicarbonate ions (Pastorekova *et al*, 2004). This reaction contributes to an acidic extracellular microenvironment and intracellular alkalosis, allowing tumour cells to survive under hypoxic conditions, favouring tumour growth, invasion, and development (Chiche *et al*, 2009).

Subsequent studies have shown that CAIX is expressed in only a few normal tissues (mainly the gastrointestinal tract), whereas it is ectopically induced under hypoxic conditions and highly overexpressed in many different cancer cell lines and tumour tissues, where *ca9* is one of the most upregulated gene in a (HIF-1)-dependent manner (Wykoff *et al*, 2000). The expression of CAIX is strongly upregulated by hypoxia and is downregulated by the wild-type von Hippel-Lindau (pVHL) tumour suppressor protein (Ohh *et al*, 2000). CAIX has already been shown to serve as a surrogate marker of hypoxia and as a prognostic indicator for many cancers (Loncaster *et al*, 2001). In some cancer cells, the *VHL* gene is mutated leading to strong upregulation of CAIX (up to 150-fold) as a consequence of constitutive HIF-1 activation (Pouyssegur *et al*, 2006). A relationship between CAIX tumour tissue expression, as detected by immunohistochemistry (IHC) and patient outcome has been reported in a wide variety of tumours (Bui *et al*, 2003; Hedley *et al*, 2003; Trastour *et al*, 2007; Choi *et al*, 2008; Klatte *et al*, 2009; Korkeila *et al*, 2009). Previous studies have reported the prognostic relevance of CAIX overexpression in NSCLC indicating a potential oncogenic function of CAIX (Swinson *et al*, 2003; Kim *et al*, 2004; Kon-no *et al*, 2006). However, these studies performed only a limiting number of IHC analyses on surgical tumour samples.

Methods that use biological materials obtained by non-invasive procedures, like plasma, can be helpful in identifying possible biomarkers for early diagnosis and in the follow-up of patients with an increased cancer risk. CAIX is a multidomain protein consisting in an N-terminal proteoglycan-like (PG) domain, an extracellular catalytic domain (CA), a transmembrane segment (TM) and a C-terminal intracytoplasmic (IC) tail (Hilvo *et al*, 2008). Recent reports have provided evidence that the extracellular domain of CAIX can be released into body fluids of patients with kidney or bladder cancers and a high CAIX plasma level was associated with poor prognosis in these patients (Zavada *et al*, 2003; Hyrsl *et al*, 2009). However, the CAIX plasma level has not yet been evaluated in patients with NSCLC.

The aim of this study was to investigate the expression of CAIX in both tissue and plasma samples from a large cohort of NSCLC patients to explore the potential role of CAIX as a prognostic biomarker for NSCLC.

## Patients and methods

### Patients

Five hundred fifty-five patients who underwent surgery for NSCLC between January 2001 and January 2008 at the Pasteur Hospital (Department of Thoracic Surgery, University of Nice, France) were included in this study. The patients received the necessary information concerning the study and consent was obtained from each of them. The study was done with the approval of the ethics committee (CHU of Nice). The main clinical and histopathological data are summarised in Table 1. Morphological classification of the tumours was assigned according to the WHO criteria (Travis *et al*, 2004). The tumours were staged according to the international tumour-node-metastasis system (Mountain, 1997). The median follow-up at the time of analysis was 35 months (3–102 months) according to the method of Schemper and Smith, (1996). Among these patients, 122 of 555 (22%) died of lung cancer.

For the ELISA assay, 209 preoperative plasma samples were obtained from these patients. The main clinical and histopathological data are summarised in Table 2. The median follow-up was 15 months (1–66 months). Among these patients 14 of 209 (8%) died of lung cancer. Plasma samples from 58 healthy individuals were used as a control for the ELISA assay.

### Tissue microarrays (TMAs) and immunohistochemistry

TMAs were constructed from archival paraffin-embedded, formalin-fixed tissue blocks. Representative tumour regions and adjacent normal lung tissue were selected for building TMAs and arrays were designed as previously described (Hofman *et al*, 2008). Immunohistochemistry for CAIX, HIF-1*α* and Ki-67 was performed on serial 4-*μ*m deparaffinised TMA sections by using an automated single-staining procedure (Benchmark XT, Ventana Medical Systems, Roche Group Inc., Tucson, AR, USA). Briefly, a rabbit polyclonal anti-CAIX antibody (clone ab15086, diluted 1 : 2000, Abcam, Cambridge, MA, USA) was added to each slide after blocking of endogenous peroxidase and proteins, and the sections were incubated with a biotinylated mouse anti-rabbit IgG (Abcam) as the secondary antibody. EDTA-pretreated sections were immunostained for HIF-1*α* (mouse, clone 54; diluted 1 : 10; BD Biosciences, Heidelberg, Germany) and Ki-67 (rabbit, clone 30–9; diluted 1 : 100; Ventana Medical Systems). Paraffin-embedded sections from healthy lung tissue exposed under normoxic/hypoxic conditions were stained for BNIP3 (rabbit, clone ANa40; diluted 1 : 250; Sigma-Aldrich, St Louis, MI, USA) and BNIP3L (rabbit, polyclonal; diluted 1 : 250, Sigma-Aldrich). 3–3′-diaminobenzidine (Sigma-Aldrich) was the chromogen used in all reactions. Positive controls for CAIX were biopsy cores of head and neck squamous cell carcinoma, which has previously been established as positive for CAIX (Koukourakis *et al*, 2001).

### Automated image analysis

After antibody staining, images were acquired using automated quantitative analysis (Hofman *et al*, 2008). The CAIX signal was measured on a grayscale of 0 (black) to 255 (white) and expressed as target signal intensity relative to plasma membrane compartment area. Grey values ranged between 0 and 152, and a value superior or equal to 50 was arbitrarily defined as CAIX overexpression. In addition, the cut-off values for the different markers were: ⩾to 35 grey levels for HIF-1*α* and ⩾10% for Ki-67. Staining intensity was based on a scale from 0 to 3 and the percentage of positive cells (0<1%, 1=1–10%, 2=10–50%, 3>50%). The product of the intensity of staining and the percentage of tumour-positive cells was then calculated to produce an IHC score of 0 to 300, as previously described (Hassan *et al*, 2008). An IHC score >40 distinguished low from high expression of CAIX (Loncaster *et al*, 2001). Nuclear staining in more than 50% of positive cells for HIF-1*α* and a labeling index of Ki-67 ⩾10% defined overexpression of both markers (Zhong *et al*, 1999; Woo *et al*, 2009). The overall score used for statistical analysis was the mean value from three spots of the same tumour. In parallel, whole-tissue sections from tumour blocks of a subset of 40 cases were stained for each marker and compared with the corresponding TMA spots using the above-mentioned scoring criteria.

### Cell culture and hypoxic exposure

The A549 lung cancer cell line (European Collection of Cell Cultures, ECACC; passage 90, Sigma-Aldrich) was maintained in RPMI 1640 supplemented with 10% FCS. Incubation in hypoxia at 1% O_2_ was carried out at 37°C in 95% humidity and 5% CO_2_/94% N_2_ in a sealed anaerobic workstation (*in vivo* 400; Ruskin Technologies, Bridgend, UK).

### Plasma CAIX ELISA immunoassay

Peripheral blood (5 ml) was taken before surgery and kept in a heparinised tube. Within 30 min of blood collection, the samples were centrifuged at 3000 r.p.m. at 4°C for 10 min to separate the plasma and blood cells. For detection of the soluble form of CAIX, we used as an internal positive control, the culture medium of A549 cells incubated in hypoxic conditions for 48 h (Ruskin Technologies).

ELISA commercial kits for the quantitative determination of plasma CAIX concentrations were used according to the manufacturer's instructions (R&D Systems, Minneapolis, MN, USA). Test samples (100 *μ*l) were pipetted into the wells and incubated for 2 h at room temperature on a horizontal orbital microplate shaker (0.12′′ orbit) set at 500±50 r.p.m. The optical density of each well was determined using a spectrophotometer microplate reader (Bioelisa-iEMS Reader MF, Logiciel Ascent Software v2.6, Labsystems, Helsinki, Finland) set to 450 nm. Results were compared with standard curves. Measurements were done in duplicate.

### Statistical methods

Analyses were performed using SPSS 16.0 statistical software (SPSS Inc., Chicago, IL, USA). We used the *χ*^2^ and Mann–Whitney tests to explore the association between tissue CAIX expression and the clinicopathological variables of patients. The Mann–Whitney and the Pearson tests were applied to assess the association between clinicopathological variables and the CAIX plasma level. The coefficient of correlation (*r*) between the expression of proteins was calculated using the Spearman's Rank test. The optimal sensitivity and specificity of plasma CAIX were determined by ROC curve analysis. In addition, the *χ*^2^ analysis was used to determine correlations between the immunohistochemical analysis of CAIX tissue tumour expression and the plasma CAIX ELISA assay. The degree of agreement between data from whole-tissue sections and the mean value of three spots was assessed using the Cohen's *κ* coefficient. Survival rates were estimated using the Kaplan–Meier method and were compared with the Log-Rank test to determine significance. The univariate and multivariate Cox proportional hazard models were used to determine the relative risk. Variables that were associated with survival with a *P*-value <0.20 in the univariate analysis were included in a multivariate regression. The variables included in the model for DSS and overall survival (OS) were pTNM stage, histological cell type and grade. All statistical tests were two-sided, and a significant *P*-value was set at the 0.05 level.

## Results

### Immunohistochemical analysis of tissue microarrays and correlation with the clinicopathological status

**Figure 1:**
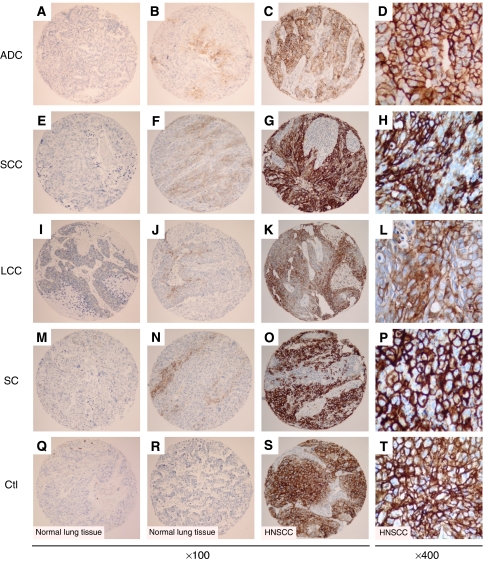
CAIX expression detected by immunohistochemistry in tissue microarray cores. Staining levels for CAIX in NSCLC histological subtypes: low (**A**, **E**, **I**, **M**), intermediate (**B**, **F**, **J**, **N**) and strong (**C**, **G**, **K**, **O**). CAIX membrane staining in adenocarcinoma (ADC) (**A**–**D**), squamous cell carcinoma (SCC) (**E**–**H**), large cell carcinoma (LCC) (**I**–**L**), and sarcomatoid carcinoma (SC) (**M**–**P**). Normal bronchial epithelium (**Q**) and alveolar tissue (**R**) are devoid of staining. *S*trong membrane staining in head and neck squamous cell carcinoma (HNSCC) cores used as positive control of CAIX immunostaining (**S**–**T**). Panels **D**, **H**, **L**, **P** and **T** are higher magnifications showing details of cells within the corresponding tumour shown on panels **C**, **G**, **K**, **O**, and **S**, respectively.

Negative or weak, intermediate, and strong CAIX membrane staining was revealed among the different NSCLC tumours (Figure 1A–P). In NSCLC the staining pattern for CAIX was membranous as observed in biopsy cores of head and neck carcinoma, used as positive control (Figure 1S–T). Tissue core biopsies taken from normal lung tissue were negative for CAIX expression (Figure 1Q–R).

Here, 135 of 555 (24.3%) tumours were designated as CAIX overexpressing (Table 1). High CAIX expression was significantly associated with histological subtypes (*P*<0.001). Sixty-eight (37%) of 184 squamous cell carcinomas and 16 (37%) of 43 large cell carcinomas overexpressed CAIX, whereas 36 (13%) of 281 adenocarcinomas overexpressed CAIX (*P*<0.001). No significant correlation was found between the CAIX status and the other clinicopathological variables (Table 1).

As for CAIX expression, the same immmunostaining intensity pattern was observed for HIF-1*α* and Ki-67 expression, which showed a nuclear staining (Supplementary Figure S1), where 133 of 555 (24%) tumours showed high HIF-1*α* immunoreactivity (Supplementary Table S1). HIF-1*α*-positive staining was associated with the histological cell type, being mainly expressed in non-adenocarcinoma subtypes (*P*<0.001) and in poorly differentiated tumours (*P*=0.0001) (Supplementary Table S1).

CAIX, HIF-1*α*, and Ki-67 expression levels observed in the TMA spots faithfully reflected the staining intensity of these proteins in whole-tissue sections from corresponding tumour blocks in a subset of 40 tumours (*κ*=0.89; data not shown).

### Association of CAIX tumour expression with hypoxia and proliferation

To assess the interrelationship between CAIX expression and hypoxia or proliferation, the CAIX expression level was compared with that of the HIF-1*α* and Ki-67 index, respectively, by using the Spearman's Rank test (Supplementary Figure S2). High CAIX expression partially correlated with high HIF-1*α* expression (*r*=0.099, *P*=0.023). In exchange, a *χ*^2^-test revealed significant association between these categorical variables (*P*=0.036). There was a strong positive correlation between the CAIX expression and the Ki-67 index (*r*=0.211, *P*<0.001).

### Plasma level of CAIX in patients with NSCLC

**Figure 2:**
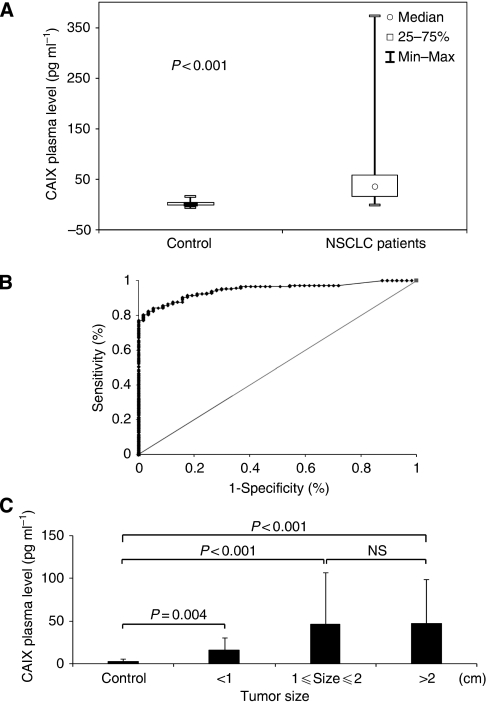
Quantitative evaluation of the plasma CAIX level by ELISA in patients with NSCLC and in healthy controls. (**A**) Box plots showing median (central dots), 25–75% quartile ranges (boxes), and minimum/maximum levels (whiskers) of plasma CAIX levels in healthy individuals (*n*=58) *vs* patients with NSCLC (*n*=209). (**B**) ROC curve analysis of CAIX as a plasma marker for NSCLC. *X* axis, 1-specificity; *y* axis, sensitivity. (**C**) Plasma concentration of CAIX according to tumour size in NSCLC and in healthy individuals. NS; non significant.

In patients with NSCLC, the mean value of CAIX in plasma was 45.40 pg/ml (range: 0–372.89 pg ml^−1^) and was significantly higher than in healthy individuals (mean=2.48 pg ml^−1^, range: 0–16.65 pg ml^−1^) (*P*<0.001) (Figure 2A).

The subtended area of the ROC curve shows a synthetic index of the overall capacity of the test in differentiating between healthy individuals and NSCLC patients (Figure 2B). The area under the ROC curve was 0.93 (CI=0.906–0.953, *P*<0.001). Using the results of the ROC curve, an analysis was made on the test performance with respect to the different threshold values, which showed that for a threshold equal to 11 pg ml^–1^, plasma; CAIX had a sensitivity of 84% and a specificity of 95% (Supplementary Table S2). Based at this cutoff we obtained a positive predictive value of 98% and a negative predictive value of 62%.

The mean value of CAIX in plasma obtained from NSCLC tumours inferior to 1 cm (*n*=29) was 28.22 pg ml^–1^ and remained significantly higher when compared with the mean value of CAIX in plasma obtained from the control group (2.48 pg ml^–1^, *n*=58, *P*=0.004) (Figure 2C).

The CAIX plasma level was significantly correlated to tumour size (*P*=0.042) (Table 2). No significant association with age, gender, smoking status, histological subtypes, or histological grade of the tumours was noted (Table 2).

### Association of the CAIX concentration in plasma with hypoxia and proliferation

The CAIX plasma level was compared with HIF-1*α* expression and the Ki-67 tumour tissues index using the Spearman's Rank test. There was no significant correlation of the CAIX plasma level with these molecules (HIF-1*α*; *r*=0.04; *P*=0.90; and Ki-67; *r*=−0.02; *P*=0.82, respectively; data not shown).

### Relationship between CAIX tumour expression and outcome of patients with NSCLC

**Figure 3:**
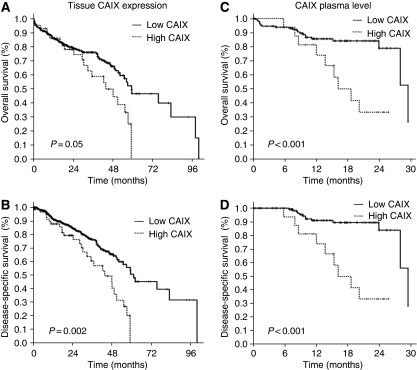
Kaplan–Meier curves of overall survival (top panels **A** and **C**), and disease-specific survival (bottom panels **B** and **D**) duration stratified according to the tissue CAIX expression detected by immunohistochemistry (left panels **A** and **B**) and the plasma CAIX levels as determined by ELISA (right panels **C** and **D**). The cut-off value for the CAIX immunostaining score was arbitrarily defined as superior or equal to 45 grey levels. The cut-off point for the CAIX plasma levels was empirically defined as superior or equal to 100 pg ml^–1^. The curves are labelled with the corresponding scores.

The status of CAIX as determined by immunohistochemistry on TMA was evaluated by Kaplan–Meier analysis for association with OS and DSS. We found that CAIX overexpression was significantly associated with shorter OS when compared with low CAIX expression levels (median survival time; 42 *vs* 55 months) (*P*=0.05) (Figure 3A). The univariate analysis revealed that CAIX overexpression was significantly associated with worse DSS (median survival time; 44 *vs* 61 months; *P*=0.002) (Figure 3B). In addition, the OS of stage I+II tumours was significantly shorter among patients overexpressing CAIX (median survival time; 44 *vs* 59 months) (*P*=0.034). However, no significant difference was noted in later stage III+IV tumours (median survival time; 32 *vs* 37 months) (*P*=0.56). The DSS of stage I+II tumours was significantly shorter for those overexpressing CAIX (median survival time; 48 *vs* 75 months) (*P*=0.001). However, no significant differences were noted in later stage III+IV tumours (median survival time; 36 *vs* 37 months) (*P*=0.290). Subsequently, a multivariate survival analysis using the Cox's proportional hazard model was performed to examine the importance of CAIX expression in patient outcome when other prognostic factors were included (Table 3). CAIX overexpression (RR=0.503, CI=0.323–0.782, *P*=0.002), the disease stage (RR=0.388, CI=0.268–0.562, *P*=0.001) and the histological grade (RR=0.611, CI=0.409–0.914, *P*=0.016) were significantly independent prognostic factors of DSS. CAIX overexpression showed a trend as an independent prognostic factor of OS (RR=0.700, CI=0.471–1.041, *P*=0.068).

With regard to the correlation between CAIX and HIF-1*α* tissue expression, the Kaplan–Meier analysis showed a trend towards worse OS and DSS for patients with both high HIF-1*α* and CAIX expression (*P*=0.087 and *P*=0.07, respectively) (Supplementary Figure S3A, B). In addition, when HIF-1*α* overexpression was evaluated by univariate analysis for a relationship to outcome according to different histological subtypes, there was a trend towards worse DSS in squamous cell carcinomas (median survival time; 52 *vs* 62 months) (*P*=0.07). In contrast, HIF-1*α* overexpression was not significantly associated with survival of NSCLC patients according to the Kaplan–Meier method or by multivariate Cox analysis (*P*=0.51; *P*=0.57, respectively) (Supplementary Figure S4, Supplementary Table S3).

### The CAIX level in plasma as a biomarker of poor prognosis in patients with NSCLC

Using an empirical thresholding method, we determined a threshold of 100 pg ml^–1^ (>90%) plasma CAIX, as an ideal cut off for stratification of patient survival. The mean plasma CAIX level >100 pg ml^–1^ was considered as a high level of CAIX, whereas that of ⩽100 pg ml^–1^ was considered as a low CAIX level. A high plasma CAIX level significantly correlated with decreased OS (*P*<0.001) (Figure 3C) and DSS (*P*<0.001) (Figure 3D). The OS of early-stage I+II tumours was significantly shorter among those with a high CAIX plasma level (*P*=0.003), but there was no significant difference in later stage III+IV tumours (*P*=0.51). In addition, the DSS of early-stage I+II tumours was significantly shorter among those with a high level of CAIX in the plasma (*P*<0.001) when compared with later stage III+IV tumours in which high CAIX was no longer associated with survival (*P*=0.23).

Multivariate analyses correlating histological subtypes, disease stage, histological grade, and a high CAIX plasma level, showed that a high CAIX plasma level was an independent prognostic factor for OS (RR=0.296, CI=0.119–0.736, *P*=0.009) and DSS (RR=0.102, CI=0.032–0.330, *P*<0.001) (Table 3).

### Expression of CAIX in tumour samples *vs* the level of CAIX in plasma

A *χ*^2^-test was used to compare CAIX immunostaining expression in tumour samples with the CAIX values in plasma from 125 patients for whom we have performed in parallel both measurements. We found no significant association (*P*=0.919) (Supplementary Table S4). Here, 90 of 125 (72%) patients had low CAIX tumour expression, but it is noteworthy that they still showed a high level of CAIX in their plasma (Supplementary Table S4). Among these patients, 28 of 90 (31%) had lymph node (pN1/pN2) or secondary metastatic sites (pM1).

## Discussion

Hypoxia is involved in biological processes promoting tumour progression and stabilises HIF-1*α*, thereby controlling expression of around a hundred genes involved in tumour metabolism, pH regulation, angiogenesis, migration, and invasion, including the membrane-associated CAIX protein (Semenza, 2003). CAIX was proposed as a surrogate marker of hypoxia and is strongly dependent on HIF-1 activation for expression (Beasley *et al*, 2001).

We showed that 135 of 555 (24.3%) NSCLC tumours overexpress CAIX. In a previous series concerning 134 lung adenocarcinomas, CAIX was overexpressed in 24.6% of cases (Kon-no *et al*, 2006). In this work, there was a significantly higher expression of CAIX in the non-adenocarcinoma histological subtypes (*P*<0.001), as described (Kim *et al*, 2005). This is probably because of the fact that SCC and LCC are frequently accompanied by necrosis and CAIX is expressed predominantly around necrotic regions. This peri-necrotic expression reflects the association of tissue tumour CAIX with hypoxia (Kim *et al*, 2004). In our study, this was supported by the positive correlation between immunostaining for CAIX and HIF-1*α* expression in tumour tissue (*P*=0.036). There was no association of the CAIX level in tumour tissues with smoking status. The occurrence of mild hypoxic condition in smokers cannot be excluded and this could be the explanation of a mechanism of adaptation, and therefore no increase in CAIX expression, because of a reduced O_2_ supply.

The interest into CAIX expression as a prognostic factor is currently expanding and targeted therapies directed specifically to CAIX are being developed (Supuran, 2008). We showed that CAIX tumour tissue expression is an independent prognostic factor for DSS in patients with NSCLC. High CAIX expression in tissues is a poor prognostic factor especially in early-stage I+II NSCLC for OS (*P*=0.034), and DSS (*P*=0.001). This is in agreement with its role in early tumourigenesis (Liao & Stanbridge, 2000). These findings are consistent with previous reports on the prognostic value of CAIX for early I/II stage resected NSCLC, in which the percentage of CAIX-positive cells was significantly associated with decreased OS (Kim *et al*, 2004). Kim *et al* (2005), reported that only a percentage of CAIX-positive cells, and not the intensity of staining, correlated with post-operative recurrence and DSS in 74 patients with early-stage NSCLC. In addition, when the perinuclear pattern of expression was considered, Swinson *et al* (2003) showed that CAIX was an independent prognostic factor for poor OS in 175 patients with NSCLC. In the same study, cytoplasmic CAIX and stromal expression were not associated with outcome, and there was only a trend towards poor prognosis for high membranous levels of CAIX in patients (Swinson *et al*, 2003). High expression of the full-length (FL) CAIX isoform was related to reduced survival of patients (Simi *et al*, 2006). Besides the expected FL mRNA, a CAIX alternative splicing isoform was detected in normal and in cancer cells, independently of the levels of hypoxia (Barathova *et al*, 2008). This alternative splicing (AS) generates a transcript lacking exons 8–9 and encodes a truncated CAIX protein lacking the transmembrane region, the intracellular tail and the C-terminal part of the catalytic domain. The expression of AS CAIX mRNA was not related to survival (Malentacchi *et al*, 2009). Overall, the antigen expression is not so homogenous and membranous CAIX expression seems to be detected more often than cytoplasmic or perinuclear CAIX expression in NSCLC. Moreover, various commercial antibodies could be the link to heteregenous patterns of CAIX expression and therefore with a great variability in terms of outcome. Although there is a general agreement that detection of CAIX by IHC could predict poor prognosis in patients with NSCLC, most studies were limited in size and had low statistical power. The differences between our study and similar previously reported IHC studies are the large number of cases and the use of a high throughput TMA analysis with automatic quantification of signals to evaluate CAIX expression in NSCLC. We strongly believe that in a large number of NSCLC cases we were able to prove that the detection of the membranous CAIX expression on TMA can be used as a poor prognostic factor in NSCLC.

In this work, HIF-1*α* positive immunolabelling significantly correlated to the histological subtype (*P*<0.001), being mostly expressed in SCC, as already reported (Swinson *et al*, 2004). It was also related to poorly differentiated tumours (*P*=0.0001), as already shown in different types of tumours (Couvelard *et al*, 2005; Trastour *et al*, 2007). These findings may reflect the existence of alternative regulatory modes of HIF-1*α*. HIF-1*α* overexpression showed a trend toward poor survival only in SCC. However, there was no significant relationship between HIF-1*α* overexpression and survival of all NSCLC patients. Our findings are supported by those of Lee *et al* (2003) who showed no association between HIF-1*α* expression and overall survival in 84 NSCLC patients. In contrast to our results, other groups reported a significant relationship between HIF-1*α* expression and shorter survival (Swinson *et al*, 2004; Kim *et al*, 2005). Swinson *et al* (2004) reported that only when a high positive cutoff ⩾60% for HIF-1*α* expression was used there was a significant relationship to poor outcome of 172 NSCLC patients. In contrast, when a cutoff ⩾ median (5%) was used as a cutoff to define negative or positive HIF-1*α* staining, it was no longer related to poor survival. Kim *et al* (2005) also reported a significant association between HIF-1*α* overexpression with a cutoff ⩾51.3% and tumour progression in a series of 74 NSCLC patients. Taken together, HIF-1*α* expression was reported to be associated with poor outcome of NSCLC patients in series that used high cut-off values and also showed a strong relationship to SCC (Swinson *et al*, 2004). These results are supported by other studies that showed a significant association between high HIF-1*α* expression and shorter survival in SCC of the head and neck (Schrijvers *et al*, 2008). However, these results are in disagreement with those of Volm & Koomagi (2000) who showed that NSCLC patients with HIF-1*α*-positive carcinomas had significantly longer median survival times than patients with HIF-1*α*-negative carcinomas. There may be methodological and biological explanations for some discrepancies in the results observed in this study. One of the issues resides in the very short half-life of HIF-1*α* (<10 min), whereas the half-life of CAIX is more than 24 h (Rafajova *et al*, 2004). The second limitation resides in the sensitivity of antibodies used for immunohistochemistry. Anti-HIF-1*α* antibodies have always been problematic, whereas the immunogenicity of the CAIX antibody is excellent with no non-specificity (Lam *et al*, 2005). Also, the variability in the results may be due to the cut-off point used to define cases as overexpressing HIF1-*α*. In this study, we have used a 45 grey-levels threshold for a semi-automated analysis corresponding to a cutoff ⩾18%. This is somewhat lower than that used in several studies showing a strong correlation with poor outcome.

One explanation for CAIX being discriminatory as a prognostic biomarker resides in the fact that all cells exposed to hypoxia, and therefore to a rapid proliferation, will become hypoxic and HIF-1*α* positive, but not necessarily CAIX positive. In many tissues, HIF-1*α* may not be sufficient to induce CAIX and only several oncogenic steps associated to epigenetic changes leading to chromatin remodeling will ‘uncover’ the *ca9* gene and make it permissive and inducible by HIF-1. We hypothesize that NSCLC in which CAIX is expressed present a more aggressive phenotype. This hypothesis is supported by our data on healthy lung tissue exposed to hypoxia (1% O_2_) for 24 h. Although hypoxia-induced HIF-1*α* stabilisation was detected by IHC on normal tissue, there was no CAIX expression (Supplementary Figure S5). Instead, we observed increased expression of BNIP3 and BNIP3L, two proteins whose expression is enhanced by HIF-1 (Guo *et al*, 2001). In this regard, further studies must be performed to check BNIP3 and BNIP3L status in the different tumour tissues. Moreover, for the subpopulation with high HIF-1*α* expression, we showed that HIF-1*α* required CAIX expression to predict a trend towards poor OS and DSS. This may suggest that expression of HIF-1*α* alone may not be enough to affect the malignant potential of all NSCLC, but rather requires the expression of HIF-1*α*-regulated genes.

Recent studies have shown that soluble CAIX is being shed from the tumour cells into the culture medium and plasma or urine of renal cancer patients (21). This corresponds to the extracellular part of the CAIX molecule, which is composed of the proteoglycan (PG)-like and CA domains that are cleaved off the plasma membrane (Zavada *et al*, 2003). Our study reports that the plasma CAIX level in NSCLC patients is significantly higher than in healthy individuals (*P*<0.001). Nevertheless, we showed that the plasma CAIX detected in patients with NSCLC is much lower than CAIX concentrations described in renal cell carcinoma, because the very high constitutive HIF-1*α* levels in these latter tumours and also differences in CAIX expression depending on the cell type (Li *et al*, 2008). Increased levels of plasma CAIX have been reported only in urological cancers (Zavada *et al*, 2003; Li *et al*, 2008; Hyrsl *et al*, 2009). Thus, our report is the first study to show evidence of high CAIX levels in the plasma of NSCLC patients. Our data show that the CAIX ELISA had a very good sensitivity (84%) and specificity (95%). Moreover, even for tumours inferior to 1 cm in size, the mean value of CAIX was significantly higher when compared with the mean value of CAIX plasma in the control group. It appears that there is no increase in CAIX in the plasma of patients bearing tumours larger than 2 cm when compared with smaller tumours (<1 cm). This is unlikely to be explained by the loss of differentiation because in our study there was no correlation with the histological grade. In the earlier stages of tumour progression, conditions such as hypoxia or ischemia may induce a high CAIX plasma level as an adaptation to confer a proliferative advantage for tumour growth and spread. However, when this malignant potential is attained in later stages of tumour growth, continued shedding of CAIX into the plasma might no longer be required. The difference in the CAIX plasma level may reflect distinct molecular pathways and genetic alterations that impact small-sized tumours, which may determine the subsequent development and risk of progression, as already suggested for bladder cancers (Turner *et al*, 2002). On the other hand, the cumulative effects of genetic lesions involved in cancer progression could alter the pathways of the hypoxic response and therefore could affect the CAIX plasma level (Rak *et al*, 2002). This is in agreement with the lack of correlation between the plasma CAIX level and Ki-67 index observed in our study. Surprisingly, the CAIX plasma level was not related to HIF-1*α* detection as observed with IHC (*r*=0.04, *P*=0.90). Moreover, there was no correlation with SCC or LCC subtypes as we observed in the case of immunodetected CAIX. With regard to the relationship between membranous CAIX and HIF-1*α* detection, this finding is raising a question on whether and how HIF-1*α* could influence the CAIX shedding from tumour cell surface.

Although our observations were inconsistent with the proposal that hypoxia and HIF-1 are the sole regulators of CAIX expression and thus shedding (Zatovicova *et al*, 2005), one cannot exclude that shedding occurs at hypoxic metastatic sites. Although the precise molecular mechanism of regulated CAIX shedding remains to be clarified, previous studies showed that dysregulation of HIF-1*α* could be caused by mutation in the von Hippel-Lindau (*VHL*) tumour suppressor gene or activation of the epidermal growth factor receptor (EGFR) (Semenza, 2003). Mutated *VHL* prevents appropriate normoxic degradation of HIF-1*α* and, as such, CAIX is highly expressed in mutated *VHL*-related tumours, independently of the extent of tumour necrosis (Swinson *et al*, 2003). *VHL* gene mutations have been previously shown in a proportion of cell lines derived from small-cell lung cancer, NSCLC, carcinoids, and mesotheliomas (Sekido *et al*, 1994). VHL mutations may be present in a subgroup of patients with negative CAIX expression in tumour tissue but with a high plasma level of CAIX. With regard to EGFR signaling, *in vitro* studies showed that signaling through the EGF pathway by phosphorylation of a cytoplasmic tyrosine residue of CAIX may either activate CAIX or enhance its expression (Dorai *et al*, 2005). In addition, phosphorylation activates phosphatidylinositol 3-kinase, resulting in phosphorylation of Akt and cancer cell survival (Dorai *et al*, 2005). This suggests that EGFR activation may also increase induction of CAIX, and therefore the shedding of the CAIX ectodomain. Moreover, the basal level of tissue CAIX may be sufficient for shedding and the soluble form of CAIX may finally represent tumour progression. Our results support this hypothesis as we showed that a high plasma CAIX level was an independent prognostic factor significantly related to worse OS and DSS of early-stage I+II NSCLC when compared with later stage III+IV NSCLC.

When the correlation between CAIX expression levels as determined by ELISA and immunohistochemistry was assessed, we did not find any relationship. This indicates that the two methods are not directly interchangeable and that their value for clinical purposes may be different. Overall, when we take into account the intratumoural heterogeneity and the different controversial results regarding the value of tissue tumour CAIX expression as a prognostic factor, we postulate that the detection of the soluble form of CAIX in plasma patients can be a more reliable prognostic factor of worse OS and DSS in patients with early-stage NSCLC.

This study shows that CAIX tumour tissue expression as detected by immunohistochemistry on TMA, can serve as an important predictor for survival in patients with NSCLC. Moreover, we showed that the plasma CAIX level is an independent prognostic factor in early-stage NSCLC. Our results support the high specificity and the potentiality of plasma CAIX as a helpful clinical biomarker for detection of NSCLC at an early stage.

## Figures and Tables

**Table 1:** Correlation of clinicopathological parameters with the CAIX expression level detected by immunohistochemistry in 555 NSCLC patients

		**CAIX status**		
**Variables**	**Overall†**	**Low†**	**High†**	***P*-value**
Patient cohort	555			
Mean age (years)‡	63.9±9.3	64.5±8.4	65.3±5.6	0.196
				
*Gender* a				
Male	415 (75)	309 (74)	106 (26)	0.439
Female	140 (25)	111 (79)	29 (21)	
				
*Smoking history* a				
Never smoked	75 (14)	54 (72)	21 (28)	0.098
Former or current smokers	480 (86)	366 (76)	114 (24)	
Tumour size (cm)‡	3.7±2.4	3.8±2.3	4.1±2.1	0.293
				
*Histological cell type* a				
Adenocarcinoma	281 (50)	245 ( 87)	36 (13)	<0.001^*^
Squamous cell carcinoma	184 (33)	116 (63)	68 (37)	
Large cell carcinoma	43 (8)	27 (63)	16 (37)	
NOS	47 (9)	32 (68)	15 (32)	
				
*pTNM stage* a				
I	278 (50)	221 (79)	57 (21)	0.676
II	103 (19)	79 (77)	24 (23)	
III	151 (27)	116 (77)	35 (23)	
IV	23 (4)	16 (70)	7 (30)	
				
*Histological grade* a				
1	214 (38)	168 (78)	46 (22)	0.785
2	187 (34)	143 (76)	44 (24)	
3	137 (25)	101 (74)	36 (26)	
4	17 (3)	13 (76)	4 (24)	
Neoadjuvant therapy^a^	67 (12)	62 (92)	5 (8)	0.105

Abbreviations: NOS = not otherwise specified; TNM = tumour node metastasis.

**†**Values expressed as n (%) or mean±s.d.

a *χ*^2^-test.

‡Mann–Whitney test.

^*^P-value significant at the 0.05 level.

**Table 2:** Correlation of clinicopathological parameters with the CAIX plasma level detected by ELISA in 209 NSCLC patients

**Variables**	**Overall†**	**Mean CAIX level (pg ml^–1^)**	***P*-value**
Patient cohort	209		
Mean age (years)^a^	64±9.18		0.172
			
*Gender*‡			
Male	143 (68)	45.73	0.290
Female	66 (32)	43.15	
			
*Smoking status*‡			
Never smoked	21 (10)	32.37	0.330
Former or current smokers	188 (90)	46.34	
Tumour size (cm)^a^	4.0±2.4		0.042^*^
			
*Histological cell type*‡			
Adenocarcinoma	109 (52)	43.63	0.280
Squamous cell carcinoma	60 (29)	51.11	
Large cell carcinoma	13 (6)	39.76	
NOS	27 (13)	37.41	
			
*pTNM stage*‡			
I	102 (49)	42.25	0.448
II	40 (19)	53.21	
III	52 (25)	43.14	
IV	15 (7)	57.19	
			
*Histological grade*‡			
1	77 (37)	50.87	0.420
2	65 (31)	41.21	
3	48 (22)	36.85	
4	19 (9)	31.32	
Neoadjuvant therapy‡	23 (11)	40.04	0.124

Abbreviations: NOS = not otherwise specified; TNM = tumour node metastasis.

**†**Values expressed as n (%) or mean±s.d.

a Pearson test.

‡Mann–Whitney test.

^*^P-value significant at the 0.05 level.

**Table 3:** Multivariate Cox regression analysis of tumour tissue and plasma CAIX levels expression for OS and DSS in patients with NSCLC

	**Overall survival**			**Disease-specific survival**		
**Variables** a	**RR**	**(95% CI)**	***P*-value**	**RR**	**(95% CI)**	***P*-value**
Histologic cell type	0.612	0.363–1.032	0.065	0.619	0.328–1.171	0.140
pTNM stage	0.424	0.314–0.572	0.001^*^	0.388	0.268–0.562	0.001^*^
Histologic grade	0.663	0.479–0.919	0.014^*^	0.611	0.409–0.914	0.016^*^
**High CAIX tissue expression**	0.700	0.471–1.041	0.068	0.503	0.323–0.782	0.002^*^
Histologic cell type	0.391	0.114–1.345	0.136	0.497	0.105–2.364	0.380
pTNM stage	0.509	0.229–1.130	0.097	0.480	0.166–1.389	0.176
Histologic grade	0.538	0.202–1.435	0.215	0.254	0.074–0.871	0.029^*^
**High plasma CAIX**	0.296	0.119–0.736	0.009^*^	0.102	0.032–0.330	<0.001^*^

Abbreviations: CI = confidence interval; TNM = tumour node metastasis.

a Coding of variables: Histological cell type was coded as 1 (ADC) and 2 (non-ADC). pTNM stage was coded 1 (stages I+II) and 2 (stages III+IV). Histological grade was coded 1 (grades 1 and 2) and 2 (grades 3 and 4).

^*^P-value significant at the 0.05 level.
