# Supplementary figures and images for: High levels of carbonic anhydrase IX in tumour tissue and plasma are biomarkers of poor prognostic in patients with non-small cell lung cancer
Source: Br J Cancer. 2010 May 11;102(11):1627–35. doi: 10.1038/sj.bjc.6605690 (PMC2883156; doi:10.1038/sj.bjc.6605690)

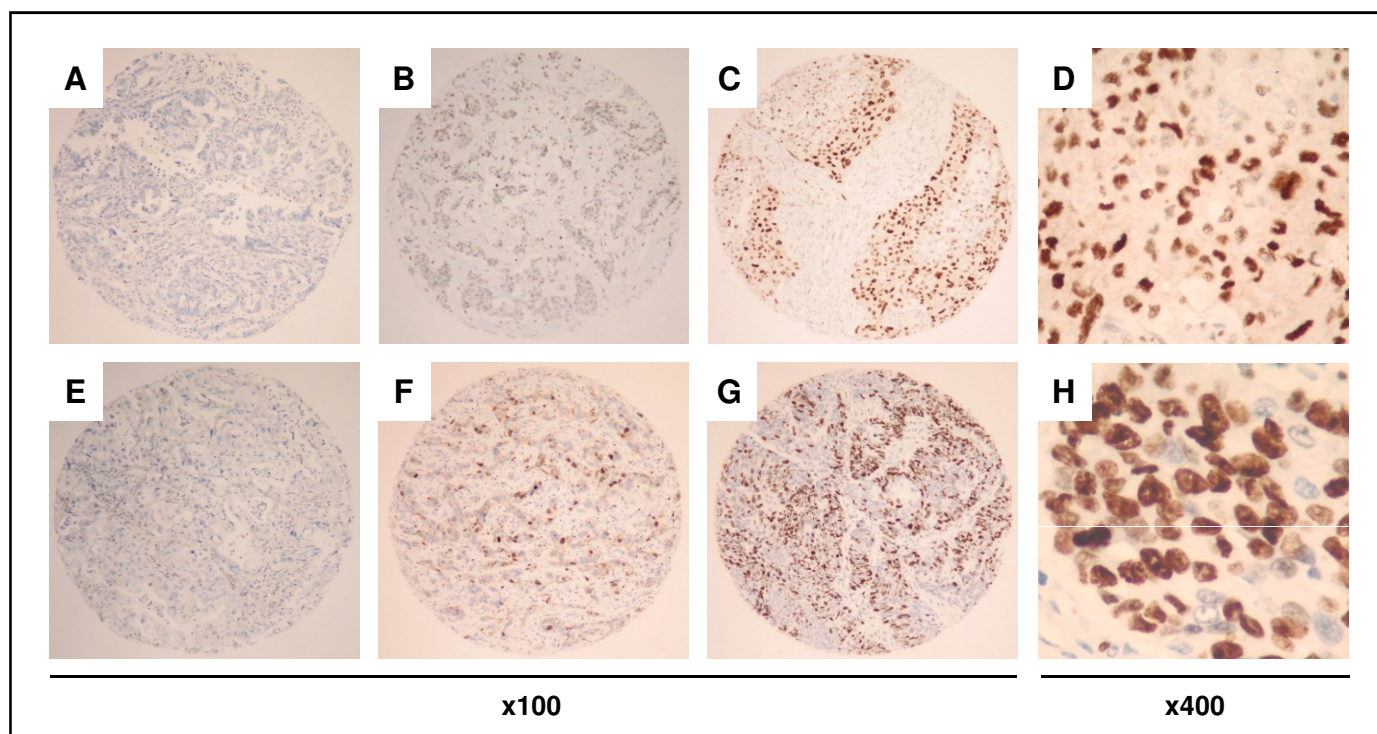

**Figure S1**  
**Ilie et al.**

Supplement: Supplementary Figure S1 [file 6605690x1.pdf]

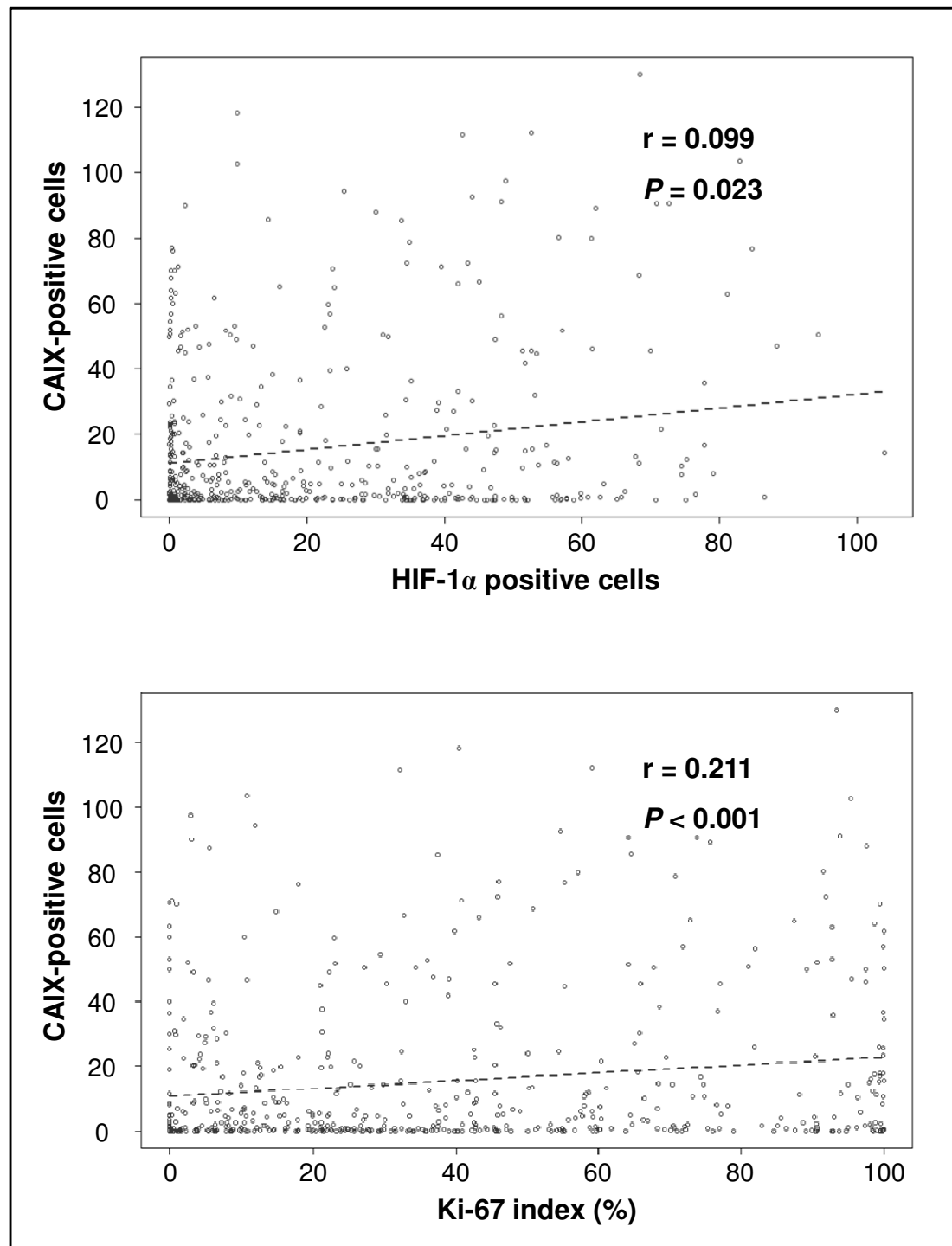

**Figure S2**

**Ilie et al.**

Supplement: Supplementary Figure S2 [file 6605690x2.pdf]

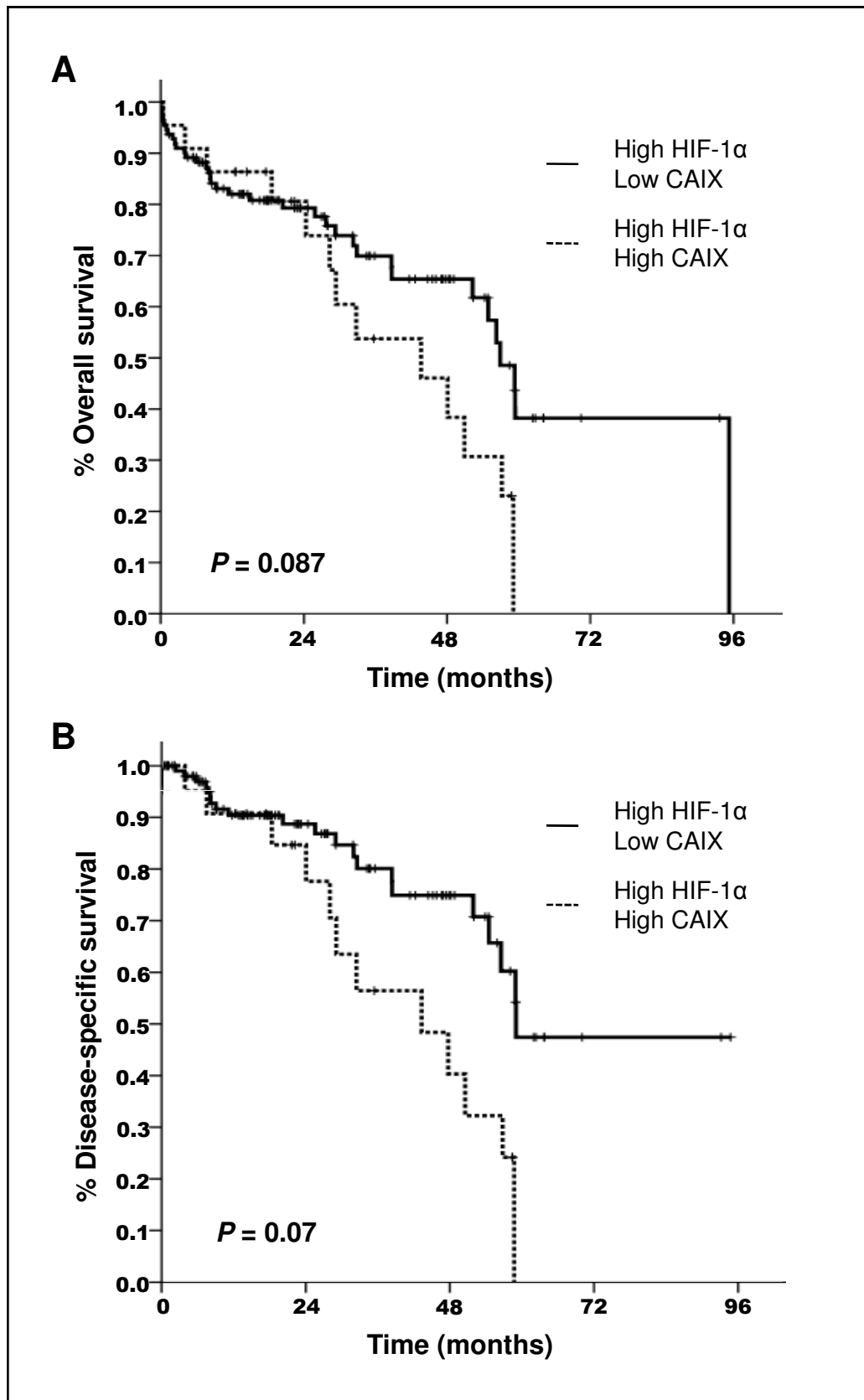

Figure S3

Ilie et al.

Supplement: Supplementary Figure S3 [file 6605690x3.pdf]

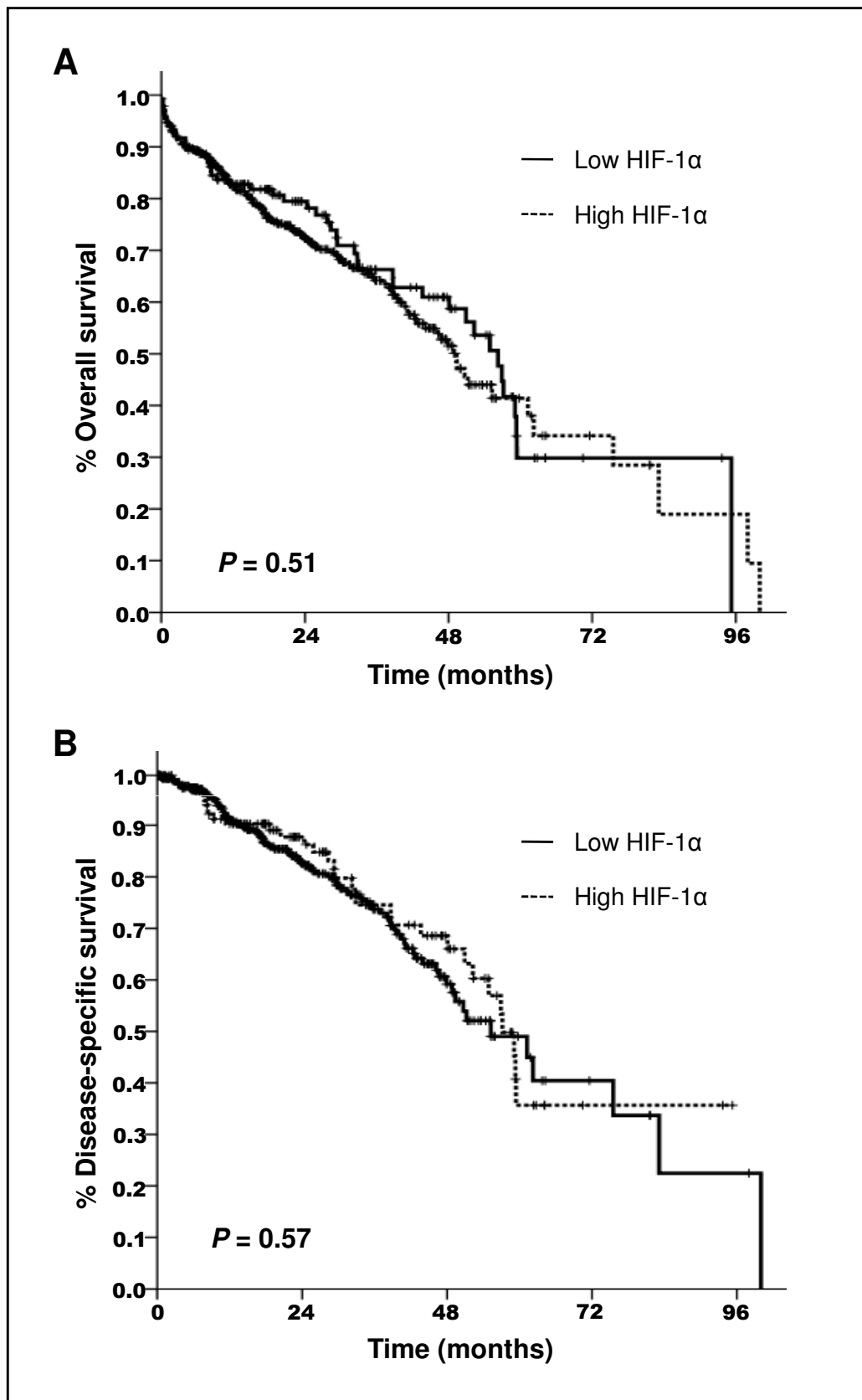

**Figure S4**

**Ilie et al.**

Supplement: Supplementary Figure S4 [file 6605690x4.pdf]

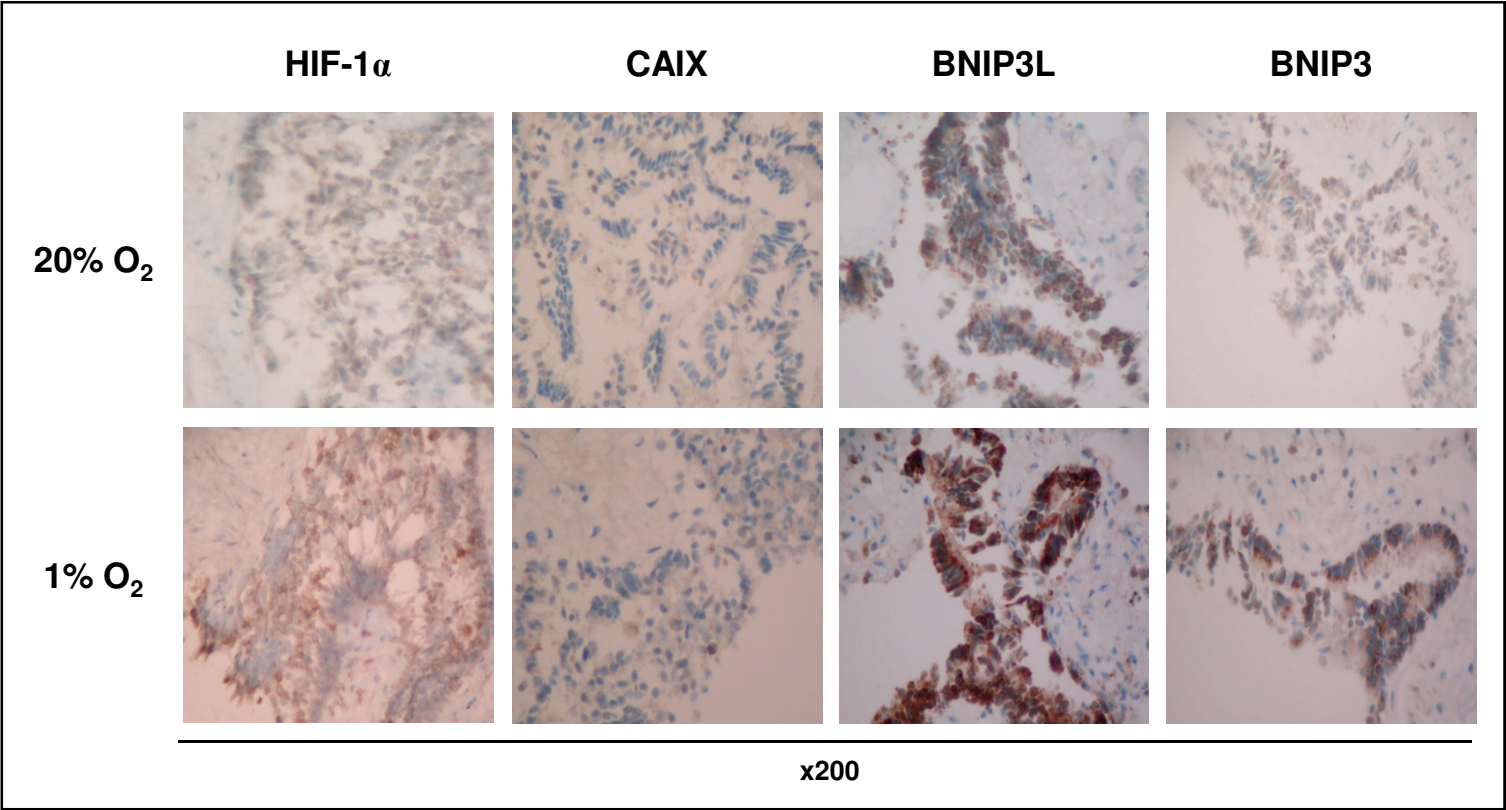

**Figure S5**  
**Ilie et al.**

Supplement: Supplementary Figure S5 [file 6605690x5.pdf]

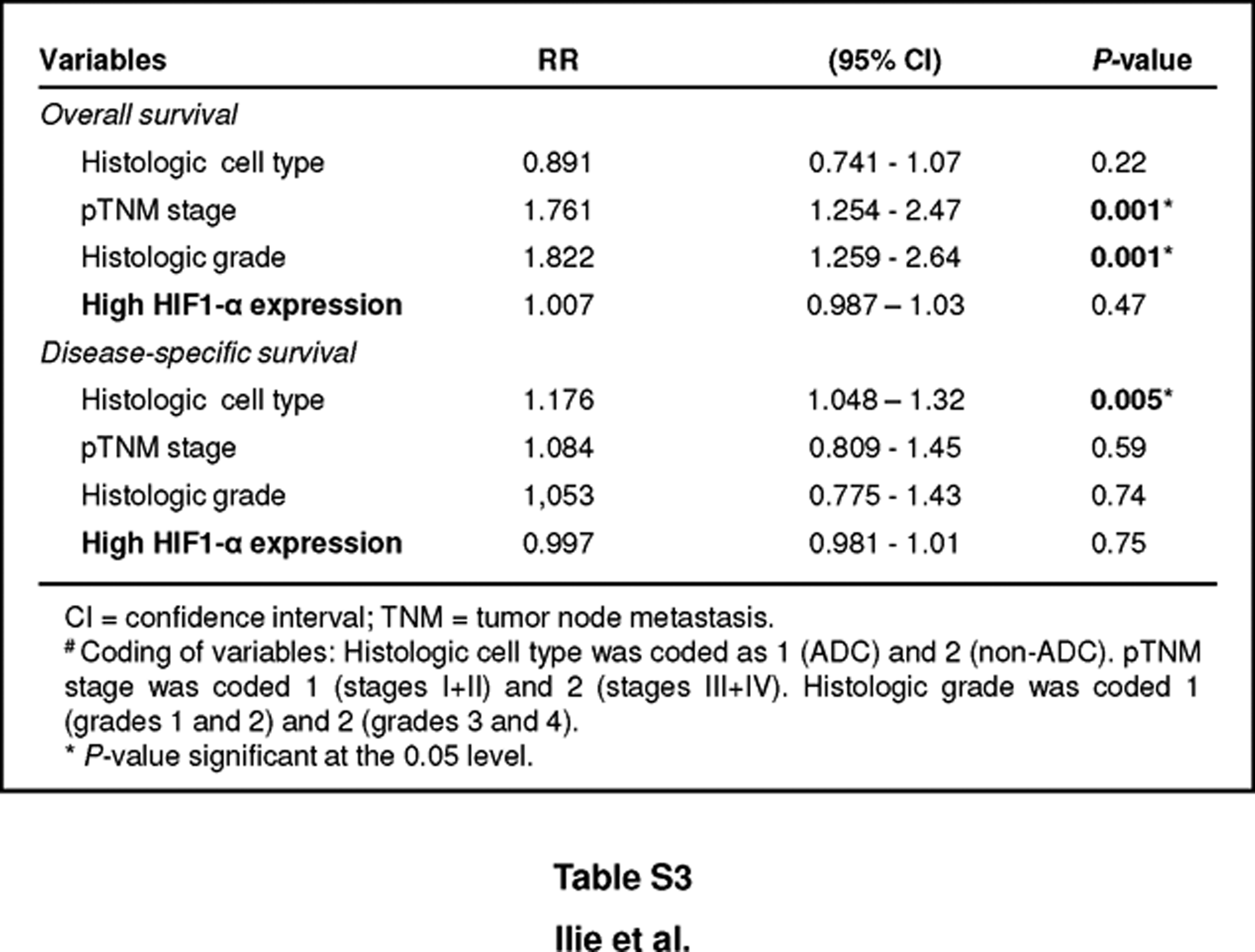

Supplement: Supplementary Table S3 [file 6605690x8.tif]
