# Supplementary material for: High levels of carbonic anhydrase IX in tumour tissue and plasma are biomarkers of poor prognostic in patients with non-small cell lung cancer
Source: Br J Cancer. 2010 May 11;102(11):1627–35. doi: 10.1038/sj.bjc.6605690 (PMC2883156; doi:10.1038/sj.bjc.6605690)
Supplement: Supplementary Table S1 [file 6605690x6.pdf]

Variables	HIF1-α status		*P*-value
	**Low** †	**High** †	
Mean age (y) ‡	65 (39, 85)	64 (32, 85)	0.370
Gender §			
Male	281 (72)	108 (28)	0.439
Female	107 (81)	25 (19)	
Smoking status §			
Never smoked	45 (82)	10 (18)	0.106
Former or current smokers	335 (74)	116 (26)	
Tumor size, median (min, max) cm ‡	3.3 (0.2, 16)	3.5 (1, 17)	0.412
Histologic cell type §			
Adenocarcinoma	236 (89)	30 (11)	**<0.001***
Squamous cell carcinoma	116 (67)	58 (33)	
Large cell carcinoma	13 (36)	23 (64)	
NOS	22 (51)	21 (49)	
pTNM stage §			
I	183 (72)	72 (28)	0.499
II	78 (75)	26 (25)	
III	111 (79)	30 (21)	
IV	16 (76)	5 (24)	
Histologic grade §			
1	151 (82)	34 (18)	**0.0001***
2	129 (74)	45 (26)	
3	91 (73)	34 (27)	
4	4 (29)	10 (71)	
Neoadjuvant therapy §	59 (88)	8 (12)	0.203

TNM = tumor node metastasis. NOS = not otherwise specified.
† Values expressed as n (%) or median.
‡ Mann-Whitney test.
§ χ^2 test.
* *P*-value significant at the 0.05 level.

Table S1

Ilie et al.
