# Supplementary material for: High levels of carbonic anhydrase IX in tumour tissue and plasma are biomarkers of poor prognostic in patients with non-small cell lung cancer
Source: Br J Cancer. 2010 May 11;102(11):1627–35. doi: 10.1038/sj.bjc.6605690 (PMC2883156; doi:10.1038/sj.bjc.6605690)
Supplement: Supplementary Table S2 [file 6605690x7.pdf]

Cut off	Sensitivity	95% CI	Specificity	95% CI	PPV	NPV
0	97.13	94.9 – 99.4	28.07	16.4 – 39.7	83.19	72.27
5	92.34	88.7 – 95.9	78.94	68.4 – 89.5	94.14	73.77
**11**	**84.21**	**79.3 – 89.2**	**94.73**	**88.9 - 100**	**98.32**	**62.07**
20	67.94	61.6 – 74.3	100	100	100	45.96

CI = confidence interval;
PPV, positive predictive value; NPV, negative predictive value.
* *P*-value significant at the 0.05 level.

Table S2

Ilie et al.
