# Supplementary material for: High levels of carbonic anhydrase IX in tumour tissue and plasma are biomarkers of poor prognostic in patients with non-small cell lung cancer
Source: Br J Cancer. 2010 May 11;102(11):1627–35. doi: 10.1038/sj.bjc.6605690 (PMC2883156; doi:10.1038/sj.bjc.6605690)
Supplement: Supplementary Table S4 [file 6605690x9.pdf]

Variables §	ELISA CAIX status		***P*-value**
	High	Low	
**IHC CAIX status**			
High	13 (10%)	2 (1.6%)	0.919
Low	90 (72%)	20 (16%)	

§ $\chi 2$ test.

Table S4

Ilie et al.
