# Supplementary material for: High levels of carbonic anhydrase IX in tumour tissue and plasma are biomarkers of poor prognostic in patients with non-small cell lung cancer
Source: Br J Cancer. 2010 May 11;102(11):1627–35. doi: 10.1038/sj.bjc.6605690 (PMC2883156; doi:10.1038/sj.bjc.6605690)
Supplement: Supplementary Legends [file 6605690x10.doc]

**Legend to supplemental tables**

**Supplementary Table S1.** Correlation of clinicopathological parameters with the HIF-1α immunostaining level in 555 NSCLC patients.

**Supplementary Table S2.** Diagnostic performance of the ELISA for CAIX based on different low CAIX plasma level cut off points.

**Supplementary Table S3.** Multivariate Cox regression analysis of tumour tissue HIF-1α expression in patients with NSCLC for overall survival and disease specific survival.

**Supplementary Table S4.** Analysis of the correlation between CAIX expression as detected by immunohistochemistry on TMA and the ELISA immunoassay in 125 NSCLC patients on which both methods were performed.

**Legend to supplemental figures**

**Supplementary Figure S1.** Staining levels for HIF-1α (A-D) and Ki-67 (E-H) in lung adenocarcinoma: low (A and E), intermediate (B and F) and strong (C and G) nuclear stainings. Panels *D* and *H* are higher magnifications showing the details of cells within the corresponding tumour shown panels C and G.

**Supplementary Figure S2.** *A*, Correlation between CAIX and HIF-1α-positive immunostaining. *B*, Correlation between CAIX-positive cells and the Ki-67 index. The Spearman’s Rank test was used. Values are expressed in grey levels.

**Supplementary Figure S3.** Kaplan-Meier curve for overall survival (A) and disease-specific survival (B) of the 133 NSCLC patients which demonstrated high HIF-1α expression dichotomized into two groups having high (n = 22) or low (n = 111) CAIX expression. The curves are labelled with the corresponding scores.

**Supplementary Figure S4.** Kaplan-Meier curves for overall survival (*A*), and disease-specific survival (*B*) of the 555 patients with NSCLC, according to HIF-1α expression. The cut off value was arbitrarily defined as superior or equal to 35 grey levels. The curves are labelled with the corresponding scores.

**Supplementary Figure S5.** Immunohistochemistry analysis of HIF-1α, CAIX, BNIP3L and BNIP3 protein expression on healthy lung tissue exposed under normoxic (20% O_2_) or hypoxic conditions (1%O_2_).
